# The influence of long distance running on sonographic joint and tendon pathology: results from a prospective study with marathon runners

**DOI:** 10.1186/s12891-016-1121-9

**Published:** 2016-07-11

**Authors:** Fabian Proft, Mathias Grunke, Christiane Reindl, Felix Mueller, Maximilian Kriegmair, Jan Leipe, Peter Weinert, Hendrik Schulze-Koops, Matthias Witt

**Affiliations:** Division of Rheumatology and Clinical Immunology, Medizinische Klinik und Poliklinik IV, University of Munich, Pettenkoferstr. 8a, D-80336 Munich, Germany; Leibniz-Rechenzentrum der Bayerischen Akademie der Wissenschaften, Garching, Germany

**Keywords:** Running, Ultrasound, Knee, Ankle, Patellar tendon

## Abstract

**Background:**

The impact of physical exercise on joints and tendons is still a matter of debate. The aim of this study was to investigate with ultrasound the acute effects of extreme physical exercise on knee and ankle joints and their surrounding structures in trained athletes.

**Methods:**

Participants of the Munich marathon were examined by arthrosonography before and after long distance running. Ultrasound assessment included grey scale and power Doppler examination of the knee and talocrural joints with surrounding tendons. Findings consistent with joint effusion, tendon and/or entheseal pathologies were documented. In addition to the ultrasound evaluation, information on training habits and past or present arthralgia or joint swelling was gathered.

**Results:**

One Hundred Five runners completed both the pre- and post-excercise ultrasound assessments (baseline and follow-up), resulting in the sonographic evaluation of 420 knee and talocrural joints. At baseline, 105 knee (50) and 38 talocrural joints (18.1) showed effusions, compared to 100 knee (47.6) and 33 talocrural joints (15.7 %) at follow-up. The differences were not significant (*p* > 0.05 each). Effusion size did not correlate with the timepoint of ultrasound assessment and was independent of covariates such as gender, age or running distance. Hypervascularity of the patellar tendon was detected in 21 cases (10.0 %) at follow-up in contrast to one at baseline (*p* < 0.001). This observation was more frequent in male than in female participants (*p* < 0.05).

**Conclusions:**

Acute physical stress is significantly associated with hypervascularity of the patellar tendon. No significant changes of synovial effusion were detected in knee and talocrural joints.

## Background

The impact of physical exercise on the morphology of joints and surrounding structures like entheses and tendons is still a matter of debate. It could be expected that physical stress acts as a stimulus on the production of synovial fluid and may provoke tendon irritation or enthesitis. However, only few studies with small numbers of subjects have dealt with this issue with conflicting results [1, 2]. Some of them found increased amounts of synovial fluid in joints of individuals who perform regular physical exercise. One trial in healthy volunteers showed an increase of joint effusions in five out of ten examined knees after physical exercise [3] and another trial showed a higher rate of ankle joint effusions after extreme physical stress in comparison to moderate sportive activity [1]. On the other hand, four magnetic resonance imaging (MRI) trials comparing the status of joints before and after a marathon competition could not demonstrate any relevant changes in the amount of synovial fluid in the hip, knee and metatarsophalangeal (MTP) joints [2, 4–6], while another study found a small increase in knee joint effusions, but no other changes in MRI imaging after a marathon race [7]. A follow-up trial after ten years of long-distance running also did not show deterioration of knee joint structures on MRI [8]. However, there are data suggesting a short and long term influence on involved tendons and entheses [1, 9, 10]. In this respect, tendons around the knees and ankles seem to be more prone to pathologies than the Achilles tendon [11, 12].

These issues are not only important in sports medicine, but also for the rheumatologist. First, many patients in whom a rheumatic condition is suspected, present to the specialist at young ages and with a background of sporting activity. Second, the enormous improvements in the treatment of rheumatic conditions have also enabled physical activity in patients with longstanding disease [13]. In both patient populations it may be difficult to distinguish the pathologic findings of the underlying disease from potential physiological alterations due to physical exercise. This may have implications for confirming a diagnosis or assessing disease activity through detection of arthritis, tenosynovitis or enthesitis.

To address these challenges, the intention of our work was to get a better understanding of the arthrosonographic changes that can be seen in individuals performing regular sporting activity and whether these increase or diminish after extreme physical exercise. To this end, we approached participants of the yearly Munich marathon and asked them to undergo an ultrasound examination and questionnaire evaluation before and after their participation. In contrast to most trials published so far, we decided to use high resolution musculoskeletal ultrasound instead of MRI as ultrasound has shown to have a comparable sensitivity and specificity [14–16].

## Methods

**Fig. 1:**
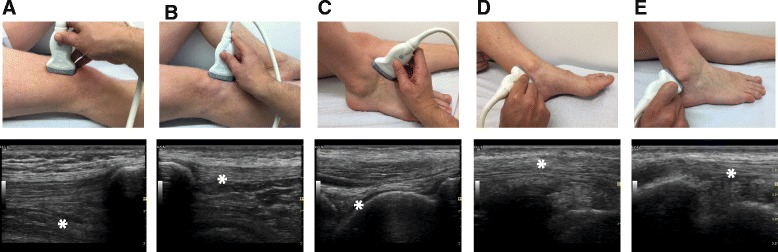
Overview of the acquired aspects with their normal ultrasound image. **a** suprapatellar longitudinal axis (* suprapatellar recessus); **b** infrapatellar longitudinal axis(* proximal patellar tendon enthesis); **c** dorsal longitudinal axis of the talocrural joint (* joint margins of the upper talocrural joint); **d** longitudinal axis of the medial ankle tendons (* posterior tibial tendon); **e** longitudinal axis of the lateral ankle tendons (* long and short peroneal tendons)

Participants of the Munich marathon, completing either the full distance of 42.195 km or the halfmarathon distance, were examined by arthrosonography before (baseline) and after the run (follow-up). The athletes were actively approached at inscription for the competition and after informed consent was obtained, a baseline ultrasound examination was perfomed 48 to 16 h prior to the start of the race. The follow-up examination was done in the recreational area of the event within two hours after the run. Ultrasound assessment included grey scale (GS) and power Doppler (PD) ultrasound examination of the knees and ankles with surrounding tendons. The ultrasound assessment was performed in a resting, lying position. At the knee, suprapatellar longitudinal and infrapatellar longitudinal aspects were acquired and assessed for presence of suprapatellar recess effusion and hypervascularity of the proximal patellar tendon enthesis, respectively. At the ankle, the longitudinal view on the talocrural joint, and longitudinal and transverse aspects of the medial, dorsal and lateral tendons were assessed for the presence of effusion (Fig. 1). Joint effusions and PD signals in both joint locations were graded semi-quantitatively on a scale ranging from 0 to 3 as described previously [17]. Findings consistent with tendon and/or entheseal pathology were graded both on a binary scale in the grey scale mode and semi-quantitatively from 0 to 3 in the power Doppler mode as described before [18]. In addition to the grading described above, a quantitative measurement of the synovial effusions in millimeters was obtained at the location of the largest diameter in both joints. For an optimal detection of fluid in the knees, subjects were asked to contract their quadriceps muscles for both the semiquantitative and quantitative assessment [19, 20].

High resolution ultrasound with GE Logiq S8 ultrasound devices was performed by three experienced ultrasound examiners (MG, FP, CR) with linear 8.5 MHz ultrasound probes. For PD examinations, the pulse repetition frequency (PRF) was set to 800 Hz. Images were electronically stored and blinded in regard to time point of acquisition and a given subject. For interpretation, the images were randomly displayed and concertedly graded by MG, CR and FP to avoid interrater and expectancy bias. Ankles and knees were read in different sets of images. In addition to the ultrasound evaluation, the participants were asked to fill out a questionnaire including demographic data such as age, gender, bodyweight, workout levels and arthralgia or joint swelling at present and in the past and were also questioned about the location of new or worsening complaints during the run.

Ultrasound findings before and after the run in different subgroups were compared with paired t-tests. Regression analysis was performed for joint effusion in knees and ankles, patellar tendon hypervascularity and the covariates height, weight, body mass index, age, gender, weekly training and running distance. For the calculations, Graph Pad Prism® software version 5.0 was used. Regression analysis and linear modeling were programmed in R software version 3.2.5 provided by the R-Foundation©. p-values of less than 0.05 were considered significant. This trial has been approved by the ethic’s committee of the University of Munich and written informed consent was obtained from all participating subjects before entering the evaluation.

## Results

### Baseline characteristics of the study population

119 runners were included in this study. While 14 participants were lost to follow-up, a total of 105 runners completed both ultrasound examinations and questionnaires at baseline and follow-up and were included in the evaluation. 70 (66.7) participants were male and 61 (58.1 %) of the participants completed the marathon distance. For the male participants, mean age, mean height and mean weight were 36 ± 12.7 years, 181 ± 7 cm and 76 ± 9.0 kg, respectively. For the female participants, mean age, mean height and mean weight were 36 ± 10.7 years, 170 ± 7 cm and 62 ± 6.5 kg, respectively. On average, the participants had completed 4.7 previous marathons and had a running experience of 7.9 years. 21.9 % of the participants completed a weekly training distance of more than 50 km, while 52.4 % ran 25 to 50 km per week and 22.9 % of the participants trainend for less than 25 km per week. An overview of the baseline characteristics of the study population is given in Table 1.

**Table 1:** Baseline characteristics of the study population

	Male female total	Male female total	Male female total
participants (%)	70 (66.7)	35 (33.3)	105 (100)
participants marathon distance (%)	47 (44.8)	14 (13.3)	61 (58.1)
participants halfmarathon distance (%)	23 (21.9)	21 (20.0)	44 (41.9)
age* (yrs)	36.4 ± 12.7	35.7 ± 10.7	36.2 ± 12.0
height* (cm)	180 ± 7	169 ± 7	177 ± 9
weight* (kg)	76.0 ± 9.0	61.6 ± 6.5	71.2 ± 10.7
BMI* (kg/m2)	23.2 ± 2.1	21.4 ± 1.7	22.6 ± 2.1
marathon finishing time* (min)	224 ± 28	263 ± 34	233 ± 34
halfmarathon finishing time* (min)	119 ± 17	127 ± 18	123 ± 18
prior marathon participations*	5.3 ± 6.9	3.5 ± 4.9	4.7 ± 6.4
weekly training distance (%)			
< 25 km	16 (22.9)	7 (20.0)	23 (21.9)
25 – 50 km	35 (50.0)	20 (57.1)	55 (52.4)
> 50 km	19 (27.1)	8 (22.8)	27 (25.7)
running experience* (yrs)	8.3 ± 7.4	7.1 ± 6.8	7.9 ± 7.3

(* = mean values with standard deviations, otherwise absolute numbers with percentages in parentheses are given. yrs = years)

### Ultrasound assessment of the knee at baseline and follow-up

A total of 210 knee joints were examined at baseline and follow-up. At baseline, an overall of 105 joints (50 %) showed an effusion, compared to 100 joints (47.6 %) at follow-up. 37 athletes (35.2 %) had no effusion, 31 (29.5 %) had unilateral effusion and 37 (35.2 %) had bilateral effusion at baseline, compared to 39 (37.1), 32 (30.5) and 34 (32.4 %) at follow-up, respectively. The differences were not significant (*p* > 0.05). The joint-specific comparison of baseline to follow-up findings showed no changes in 140 knee joints (66.7 %) whereas an alteration was detected in 70 knee joints (33.3 %). 32 knees showed new or progressive effusions and 38 knees showed less or disappearence of effusion at follow-up. In regard to the mean effusion size, there were no significant changes between baseline and follow-up with 15.5 mm at baseline and 13.9 mm at follow-up (Table 2).

**Table 2:** Results summary of ultrasound findings before (baseline) and after running (follow-up)

	Baseline follow	Up p	Baseline follow
Knee joints with suprapatellar effusion (%)	105 (50.0)	100 (47.6)	0.696
Mean amount of suprapatellar effusion (mm with SD)	15.5 ± 17.0	13.9 ± 15.9	0.151
Knee joint baseline-to-follow-up comparison (%)			
unchanged joint finding at follow-up		140 (66.7)	
deteriorated joint finding at follow-up		32 (15.2)	
improved joint finding at follow-up		38 (18.1)	
Prevalence of patellar tendon hypervascularity (%)	1 (0.5)	21 (10.0)	< 0.001
Talocrural joints with effusion (%)	38 (18.1)	33 (15.7)	0.603
Mean amount of talocrural effusion (mm with SD)	5.2 ± 11.8	4.7 ± 11.6	0.565
Talocrural joint baseline-to-follow-up comparison (%)			
unchanged joint finding at follow-up		173 (82.3)	
deteriorated joint finding at follow-up		15 (7.2)	
improved joint finding at follow-up		22 (10.5)	

Concerning the assessment of the patellar tendon, hypervascularity of the proximal enthesis was detected in a total of 21 sites (10.0 %) by power Doppler at follow-up. One finding was already present in one athlete at baseline while new findings in this location were present in 20 examinations after the run. Unilateral respective bilateral patellar tendon hypervascularity was found in 13 (12.4) and 4 (3.8 %) athletes. The difference between baseline and follow-up was significant (*p* < 0.001) and remained significant in the male subgroup and in the subgroup of participants younger than 35 years. No significant differences between baseline and follow-up were found in female participants, participants older than 35 years and the other remaining subgroups (Table 3).

**Table 3:** Results summary for patellar tendon findings before (baseline) and after running (follow-up)

Prevalence of patellar tendon hypervascularity (%)	Baseline	Follow-up	*p*
total	1 (0.5)	21 (10.0)	**< 0.001**
male participants	1 (0.7)	18 (12.8)	**< 0.001**
female participants	0 (0)	3 (4.3)	0.245
BMI < 23 kg/m2	0 (0)	9 (7.3)	**0.003**
BMI > 23 kg/m2	1 (1.2)	12 (14.3)	**0.002**
age ≤ 35 years	0 (0)	15 (13.2)	**< 0.001**
age > 35 years	1 (1.0)	6 (6.3)	0.118
weekly training < 50 km	1 (0.6)	13 (9.1)	**0.002**
weekly training > 50 km	0 (0)	8 (14.8)	**< 0.001**
half marathon distance	0 (0)	7 (8.0)	**0.014**
marathon distance	1 (0.8)	14 (11.5)	**< 0.001**

*p* values < 0.05 were considered significant and are highlighted in bold print

### Ultrasound assessment of the talocrural joint at baseline and follow-up

A total of 210 talocrural joints were examined at baseline and follow-up. In 38 joints (18.1 %) findings consistent with joint effusion were detected at baseline, compared to 33 joints (15.7 %) at follow-up. 79 athletes (75.2) had no effusion, 14 (13.3) had unilateral effusion and 12 (11.4 %) had bilateral effusion at baseline, compared to 82 (78.1), 12 (11.4) and 10 (10.5 %) at follow-up, respectively. The differences were not significant (*p* > 0.05). The joint-specific comparison of baseline to follow-up findings showed no changes in 173 talocrural joints (82.3 %), whereas an alteration was detected in 37. 15 joints showed new or progressive effusions and 22 joints showed less or disappearence of effusions at follow-up. In regard to the mean effusion sizes, there were no significant changes between baseline and follow-up with 5.2 mm at baseline and 4.7 mm at follow-up (Table 2). Concerning the tibial and peroneal assessements of the ankle tendons, neither tendon sheath effusions nor hypervascularities were detected at baseline or follow-up on either side.

### Pearson’s correlation analysis for joint effusion, patellar tendon hypervascularity and covariates

**Fig. 2:**
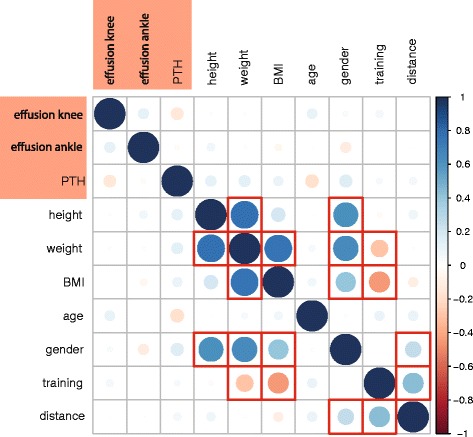
Correlation matrix of effusion in knee and ankle, patellar tendon hypervascularity and covariates. Positive correlations in blue, negative correlations in red, size of circles illustrates strength of correlation. Significant correlations framed in red (PTH - patellar tendon hypervascularity, BMI - body mass index)

The size of joint effusion of knee and talocrural joints did not correlate with the time point of assessment, i.e. pre- versus post-running. Changes of joint effusion of knee and talocrural joints and of patellar tendon hypervascularity correlated weakly but significantly with changes of the respective contralateral side (r = 0.33, *p* = 0.007, r = 0.40, *p* < 0.001, and r = 0.34, *p* = 0.001, respectively). No significant correlation was found for changes of joint effusion of the knee with changes of joint effusion of the dependent talocrural joint or vice versa (Fig. 2).

In addition, the following significant correlations were found: presence of joint effusion of knee or talocrural joints at baseline with the presence of joint effusion of the contralateral side at baseline (r = 0.47, *p* < 0.001, and r = 0.56, *p* < 0.001, respectively), and presence of joint effusion of knee or talocrural joints at baseline with joint effusion on the same side at follow-up (0.45, *p* < 0.001, and r = 0.43, *p* < 0.001, respectively). The covariates height, weight, BMI, age, gender, training and running distance did not correlate with changes or presence of joint effusion in knee or talocrual joints nor did they correlate with patellar tendon hypervascularity. For the covariates, the following correlations were found: gender correlated with weight, height, BMI and running distance, training was inversely correlated with weight and BMI and was positively correlated with running distance (Fig. 2).

## Discussion

The data presented here were gathered during the yearly Munich marathon race with more than 14.000 participants. In contrast to most trials published so far, we decided to use high resolution musculoskeletal ultrasound instead of MRI, given the fact that ultrasound has shown to have a comparable sensitivity and specificity [14–16, 21]. For our purpose, the advantage of this approach is obvious as ultrasound is easily available, quick to perform, and can be moved to the location where test subjects can be reached. These advantages resulted in the recruitment of more than 100 individuals, which to our knowledge has not been reached by other similar trials performed so far.

According to our data, extreme physical stress in highly trained athletes does not influence developement or change of joint effusion in the knee or ankle. This does not differ between male and female participants and was also independent from other covariates such as body-mass-index, age, average weekly training and whether the full or the half marathon distance was covered (Table 2 and Fig. 2). As the follow-up examination was performed within two hours after the race without further follow-up examinations, it can be argued that we could demonstrate only short term effects of extreme exercise on knee and ankle joints. However, we also analysed whether joint effusions at baseline depend on the mean weekly training of the participants but we could not find any significant differences (data not shown). We therefore conclude that there seems to be neither a long term effect of regular sportive activity nor a short term effect of extreme physical stress on the amount of effusion in the respective joints examined in this study.

This however was different for the ultrasound assessment of the patellar tendon, where we could detect significantly more runners with increase of patellar tendon hypervascularity at follow-up. The difference was preserved in the male subgroup and also in most of the other subgroups analysed, identifying acute physical stress as the main contributor to the observed patellar tendon changes (Tables 2 and 3). In contrast to the patellar tendon, the tendons of the anterior tibial, posterior tibial and peroneal aspects of the ankle did not show findings of hypervascularity or tendon sheath effusions before or after the race. Moreover, the ultrasonographic findings were not associated with joint or tendon pain as reported by the participants (data not shown).

Our results partly confirm previously published data of smaller studies. In knee joints of eight runners for example, Hohmann et al. could not demonstrate bone marrow edema, periosteal stress reactions or joint effusions on MRI images after exercise and hence concluded that physical stress in long-distance running is well tolerated [5]. Another study using MRI of the knees before, immediatly after and up to eight weeks after a marathon performance also could not detect negative long-term-effects of physical stress [8]. In contrast to our results, some studies including talocrural joints were able to detect post-exercise findings in this location. Lohmann et al., for instance, demonstrated an increased amount of joint fluid in the talocrural joint. Another study demonstrated the presence of post-exercise bone marrow edema [1, 22] which of course cannot be shown by ultrasound.

Concerning the patellar tendon, ultrasound findings were described in studies involving athletes such as rugby or soccer players and ballet dancers and included microcalculi, thickened tendons, areas of cystic degenerative change, macrocalculi and neovascularisation [23, 24]. Of note, these studies did not compare pre- and post-exercise findings but rather assessed chronic tendon damage while we were interested in acute exercise-related tendon alterations. Considering the given time frame, we found power Doppler ultrasound to be a valid method for this purpose. One small study that did pre versus post exercise tendon assessements including the patellar tendon found abnormalities such as infrapatellar bursitis or proximal patellar ligament thickening after only mild exercise, but power Doppler was not assessed [9]. Kubo et al. were able to demonstrate changes in the blood circulation of the patellar tendon during exercise which appeared to be related to the plasticity of the tendon’s mechanical properties, a finding that was different from the Achilles’ tendon in that study [11, 25]. One large study performing ultrasound assessments of 95 semiprofessional badminton players found that hypervascularity was present in a high percentage of examined tendons. No significant association to pain was observed but the findings significantly decreased during the active season of the players [10]. Other studies focused on parameters such as the cross-sectional diameter of the patellar tendon rather than power Doppler and detected an increased thickening of the tendon diameter after exercise [26, 27].

There are some noteworthy limitations of our study. First, selection bias can not be excluded considering we assessed 105 out of 14.000 potential participants, but recruitement of more participants would not have been feasible with our ressources. Still, compared to other studies on the topic, we present the largest cohort investigated so far. By actively approaching the participants on a random basis, we are confident, that a selection bias was excluded as far as possible. In addition, presence of joint effusion in both knee and ankle joints at baseline and follow-up correlated with presence of joint effusion of the respective contralateral joint, which may indicate expectancy bias during ultrasound assessment. Although we analysed multiple covariates to account for confounders as much as possible, we can not exclude that additional factors may contribute to non-independence of our observations. Also, the observation that there seemed a trend to less effusion after running may be attributed to different positioning of the probe and inter-reader variability. Further, while we used an ultrasound-based approach to the issue, some other studies were performed with MRI, so caution must be given when comparing results of studies using different methods. However, our main intention as rheumatologists was to clarify whether extensive physical stress influences our determinants for joint and tendon abnormalities, for which ultrasound is an evidential method [17, 18, 28]. Furthermore, the examination of structures like the Achilles‘ tendon, the hip joint or the metatarsophalangeal joints would have been interesting as well, but for these examinations, a relocation of the participants respective a switching of the ultrasound probe would have been necessary, both of which would not have allowed us to investigate the prospected number of subjects in the given time frame. Finally, the question how long the patellar hyperperfusion persists after the acute stress is interesting, but sonographic follow-up examinations were not performed because it was not feasible with our study design. Therefore, we cannot premise that the patellar hypervascularity that we visualize by ultrasound is pathological or just a physiologic reaction to extreme physical stress. The data from Boesen et al. would argue in favor of the latter assumption [10].

## Conclusion

As a key point for sports medicine experts, rheumatologists, orthopedics and radiologists, our observations may help to better understand arthrosonographic findings in physically active subjects. According to our data, effusions in knee and talocrural joints may not be attributed to regular or acute physical activity. On the other hand, caution should be given to define ultrasonographic findings of the patellar tendon as pathologic or disease related in those who have performed extensive physical activity before the ultrasound assessment. With 105 evaluable study subjects, we are confident that our data can substantially contribute to previous observations with partially conflicting results.

## Abbreviations

BMI, Body mass index; GS, Greyscale; MRI, Magnetic resonance imaging; MTP, Metatarsophalangeal; PD, Power doppler
