# Erratum to: The influence of long distance running on sonographic joint and tendon pathology: results from a prospective study with marathon runners

**DOI:** 10.1186/s12891-016-1223-4

**Published:** 2016-09-02

**Authors:** Fabian Proft, Mathias Grunke, Christiane Reindl, Markus A. Schramm, Felix Mueller, Maximilian Kriegmair, Jan Leipe, Peter Weinert, Hendrik Schulze-Koops, Matthias Witt

**Affiliations:** 1 Division of Rheumatology and Clinical Immunology, Medizinische Klinik und Poliklinik IV, University of Munich, Pettenkoferstr. 8a, D-80336 Munich, Germany; 2 Leibniz-Rechenzentrum der Bayerischen Akademie der Wissenschaften, Garching, Germany

## Erratum

After publication of the original article [1], we realised that we had failed to include the name of one of our co-authors, Markus A. Schramm, who had contributed substantially to the acquisition of the study data and was also involved in drafting the manuscript. His name was omitted in error and all of the original co-authors agree with the proposed change.

The correct author list should be as follows:

Fabian Proft, Mathias Grunke, Christiane Reindl, Markus A. Schramm, Felix Mueller, Maximilian Kriegmair, Jan Leipe, Peter Weinert, Hendrik Schulze-Koops and Matthias Witt

The Authors’ contributions section of the article should now read as follows:

FP, MG and MW designed the study, analyzed and interpreted the data and drafted the manuscript. CR, MAS, FM, MK and JL acquired the data and helped to draft the manuscript. HSK drafted and critically revised the manuscript. PW analyzed and interpreted the data of the regression analysis. All authors read and approved the final manuscript.

No changes to the Competing interests and Acknowledgements sections are required as a result of the addition of Markus A. Schramm to the authorship.
